# Using Metrics to Describe the Participative Stances of Members Within Discussion Forums

**DOI:** 10.2196/jmir.1591

**Published:** 2011-01-10

**Authors:** Ray Jones, Siobhan Sharkey, Janet Smithson, Tamsin Ford, Tobit Emmens, Elaine Hewis, Bryony Sheaves, Christabel Owens

**Affiliations:** ^5^Independent Expert by ExperienceExeterUnited Kingdom; ^4^University of ExeterExeterUnited Kingdom; ^3^Peninsula Medical SchoolUniversity of ExeterExeterUnited Kingdom; ^2^Devon Partnership NHS TrustWonford HouseDryden RoadExeterUnited Kingdom; ^1^Faculty of HealthUniversity of PlymouthPlymouthUnited Kingdom

**Keywords:** Online communities, metrics, discussion forums, self-harm, moderation, participative stance

## Abstract

**Background:**

Researchers using forums and online focus groups need to ensure they are safe and need tools to make best use of the data. We explored the use of metrics that would allow better forum management and more effective analysis of participant contributions.

**Objective:**

To report retrospectively calculated metrics from self-harm discussion forums and to assess whether metrics add to other methods such as discourse analysis. We asked (1) which metrics are most useful to compare and manage forums, and (2) how metrics can be used to identify the participative stances of members to help manage discussion forums.

**Methods:**

We studied the use of metrics in discussion forums on self-harm. *SharpTalk* comprised five discussion forums, all using the same software but with different forum compositions. *SharpTalk* forums were similar to most moderated forums but combined support and general social chat with online focus groups discussing issues on self-harm. Routinely recorded time-stamp data were used to derive metrics of episodes, time online, pages read, and postings. We compared metrics from the forums with views from discussion threads and from moderators. We identified patterns of participants’ online behavior by plotting scattergrams and identifying outliers and clusters within different metrics.

**Results:**

In comparing forums, important metrics seem to be number of participants, number of active participants, total time of all participants logged on in each 24 hours, and total number of postings by all participants in 24 hours. In examining participative stances, the important metrics were individuals’ time logged per 24 hours, number of episodes, mean length of episodes, number of postings per 24 hours, and location within the forum of those postings. Metric scattergrams identified several participative stances: (1) the “caretaker,” who was “always around,” logged on for a much greater time than most other participants, posting but mainly in response to others and rarely initiating threads, (2) the “butterfly,” who “flitted in and out,” had a large number of short episodes, (3) two “discussants,” who initiated many more discussion threads than anybody else and posted proportionately less in the support room, (4) “here for you,” who posted frequently in the support room in response to other participants’ threads, and (5) seven “people in distress,” who posted many comments in the support room in comparison with their total postings and tended to post on their own threads.

**Conclusions:**

Real-time metrics may be useful: (1) by offering additional ways of comparing different discussion forums helping with their management, and (2) by identifying participative stances of individuals so allowing better moderation and support of forums, and more effective use of the data collected. For this to happen, researchers need to publish metrics for their discussion forums and software developers need to offer more real-time metrics facilities.

## Introduction

Many health-related discussion forums combine the roles of supporting their members while offering the possibility of discussing general issues. The emphasis between focus group discussion and mutual support may vary. For vulnerable groups such as young people who self-harm (YPSH) the support element may be very important. If discussion forums have formal research aims, then the way participants contribute may be as important as the content of the discussion. In either case, moderators and forum owners may have to make decisions about the safety of continuing a forum and about the management of the forum. This study retrospectively explored the use of metrics, asking whether they might be useful in the management of a forum or in the analysis of contributions to a discussion.

In 2001, Preece [1] argued that “Little attention has focused so far on evaluating the success of online communities.” She suggested various metrics such as the number of participants in a community, the number of messages per unit of time, members’ satisfaction, and some less obvious measures such as amount of reciprocity, the number of on-topic messages, trustworthiness, and several others, but warned that these should be triangulated with qualitative data. In 2004, Phippen [2] suggested that the evaluation of virtual community usage and user behavior had its roots in social science approaches such as interview, document analysis, and survey, but that little evaluation had been carried out using traffic or protocol analysis. Since then Web analytics has gained huge commercial importance with methods such as Google Analytics having global use [3-5]. Although Syme [6] argues that “metrics for social media is in its infancy stage,” much has been written about social networking metrics. For example, a case study [7] of the analysis of 10 months’ Facebook data for UNICEF-USA assessed the impact of their efforts to get users to make online contributions. This included analysis of metrics such as visitor sessions, unique visitors, click-throughs to the main site, and percentage of the traffic on the main site generated by Facebook. In their study the key metric was the rate (1.8%) of conversion from Facebook visitor to donor (the key goal for UNICEF). Although similar to studies measuring the use of social networking for marketing, and those using metrics to gain insight into the health status of whole online populations [8,9], our study was concerned with the facilitation of an online focus group discussion within a safe environment.

Most studies of online communities tend to take a qualitative approach or use surveys among users (eg, [10-13]), although some have used a combined approach. For example, Rao et al [14] classified participants as lurkers or posters according to metrics and then used survey methods. Toral et al [15,16] used social network analysis to explore social interactions in a task-oriented community of Linux users. They included the use of various network maps and use of the Gini coefficient. The Gini coefficient is a measure of dispersion more usually known in presentations of inequalities in income, but Toral et al [16] used it to describe inequalities in contribution to a discussion forum.

Can metrics help us compare one discussion forum with another, and do they add to what can be found using other methods of forum analysis such as online surveys, and thematic or discourse analysis? Strijbos et al examined roles and participative stances in the context of collaborative learning [17-20] mainly using qualitative methods. Can metrics tell us anything new about forum participants? If so, should different metrics be made easily accessible to allow moderators and forum owners to monitor and adapt their forums in real time?

The aim of this study was to examine and report metrics in five different versions of an online forum on self-harm, and to assess their usefulness for (1) describing and comparing forums and (2) describing the participative stances of individuals within forums.

Metrics are likely to depend on how and why a forum was set up, and its interface, functionality, and size. We compared five different forums, all with the same interface and purpose, set up as part of a single project on self-harm, known as *SharpTalk*. If metrics from these five forums help to explain our findings from other methods of analysis, they may have wider use in comparison of discussion forums or as a moderation tool.

## Methods

### Setting

#### SharpTalk

The *SharpTalk* project [21] was set up to explore the potential of online communities to facilitate engagement and shared learning between health care professionals and YPSH. We used the forum as an online focus group [22-24] to observe how health care professionals and YPSH interacted and to provide a supportive online environment for the duration of the study (final report available from authors).

#### Recruitment

Announcements on existing online self-harm forums were used to recruit 77 YPSH. We recruited 18 National Health Service (NHS) professionals and final-year students in health/social care disciplines by emails and advertisements in two universities, three NHS Trusts, and on the national websites of relevant professional bodies. One researcher (SS) was responsible for email contact. All participants were anonymous and known only by a chosen username.

#### Forums

Participants were initially allocated to one of three separate forums, made up as follows (phase 1):

- Forum 1: 34 YPSH
- Forum 2: 26 YPSH + 5 health care professionals
- Forum 3: 17 YPSH + 13 health care professionals.

Each forum had three rooms: support/crisis, discussion/debate, random/off-topic.

#### Team Roles

Six of the authors (EH, RJ, TE, JS, BS, TF) acted as moderators, while two (SS, CO) were known as researchers and introduced topics and facilitated discussions.

#### Reconfiguration of the Forums

By the start of the third week, only two health care professionals had posted more than once; 12 out of 18 had not posted at all. The third forum therefore had few active members and little support was available for those in crisis. Although the moderators were offering support and taking on a more extensive role than simple policing, it was felt that the situation was not safe for participants. Following consultation with *SharpTalk* participants, the Ethics Committee, and funders, we therefore reconfigured the forum compositions, reallocating all participants to two instead of three forums with the aim of achieving a more even distribution of active participants (phase 2). These were made up as follows:

- Forum 4: 39 YPSH + 6 health care professionals
- Forum 5: 38 YPSH + 12 health care professionals.

These two discussion forums ran for a further 10 weeks, until research and funding considerations required them to end.

#### Forum Characteristics

As in most discussion forums, participants could see who else was online in their forum. There was also a private messaging facility. Participants could post or respond to messages at any time and were encouraged to post on-topic for the relevant room. Posts were saved with time and date.

#### Differences Between SharpTalk and Other Discussion Forums

*SharpTalk* was set up to explore whether and how health care professionals and YPSH would interact online. It therefore combined peer support and general social chat with focused discussion and debate. Discussion topics were introduced by the researchers, as in an online focus group, or by participants, or sometimes by moderators. The failure of health care professionals to participate actively in the forum resulted in the moderators taking on a much more involved role than is usual, acting almost like proxy health care professionals.

### Metrics and Other Data

#### Data Recorded by the Forum Software

The forum software recorded data in four sequential files. (1) Pages viewed: each record comprised a time stamp, user ID, page code, and URL for every page viewed by users. Pages included menus as well as messages, so the data provide an estimate of activity rather than an exact count of messages viewed. (2) New nodes: information on each thread or node was recorded as it was started (node ID, node title, user ID, name, and time stamp). (3) Postings: each record comprised a posting ID, node ID, user ID and name, user name, the actual post, and time stamp. (4) Users’ file: user ID, user name, and forum.

#### Derived Data

The four source files listed above were merged and manipulated to derive other data such as episodes (Table 1). An episode was defined as a period in which the participant’s name is showing on the logged-on list. In these forums, users who did not look at a new page for 15 minutes were removed from the logged-on list; when they started looking again it was counted as a new episode. Episodes were not specifically recorded but were imputed from the time between time stamps on page views. By examining sequential time stamps for individuals from the pages-viewed file, if a gap of more than 15 minutes was found, or a time stamp was the last page viewed, we assumed an end of episode (rounded up by 1 minute). Once the episodes were identified we calculated the length of episodes in minutes. Staff (moderators and researchers) had access to all forums and could move from one to another. As a result, it was not possible to allocate staff viewings and postings to specific forums.

**Table 1:** Metric definitions

Metric group	Definition	Source or definition
Participants	(1) Total number of participants, (2) number of active participants (ie, those who at some time logged onto the discussion forum), (3) number of participants who never posted	Total number from those registered on forum; active participants from user IDs listed at least once on pages-viewed file; those who never posted from users who never had ID listed in postings file
Episodes	(1) Total number of participant episodes, (2) total number of participant episodes per 24 hours, (3) mean number of participant episodes per 24 hours, (4) average length of episodes	Episodes were imputed from time between time stamps on pages-viewed file, to correspond with the way the forum software removed a participant’s name from the logged-on list if the participant did not actively do something for 15 minutes; 24-hour metrics were based on whole calendar days from recorded data
Time	(1) Total minutes logged on, (2) total participant minutes per 24 hours, (3) mean minutes per participant per 24 hours	The total minutes logged on was the sum of the episodes; 24-hour metric based on whole calendar days
Posting	(1) Number of messages posted per forum per 24 hours, (2) number of messages posted per person per 24 hours, (3) for individuals, location of postings (own thread, somebody else’s thread), (4) for individuals, location of postings (different sections of the forums: support room, discussion room, random/off-topic room)	Number of postings from postings file, analyzed by date, forum, and individual; location of postings derived by combining new nodes file (node ownership) and postings files
Reading	(1) Number of pages viewed per forum per 24 hours, (2) number of pages viewed per person per 24 hours	Derived from pages-viewed file; a page might be either an index (ie, list) of message headings, or the actual message

#### Other Sources of Data

As part of the registration process, all participants completed an online questionnaire that included demographics and information on Internet use and self-harming behavior. In the last few weeks of the forums, all participants were invited to give their views by rating statements about *SharpTalk* or about discussion forums in general. Finally, we have the views of participants, moderators, and researchers as recorded in the discussion forum messages.

### Analysis

#### A Priori Hypothesis

Our hypothesis was that forum 3 would show significantly less activity than forums 1 and 2, and that there would be no difference between forums 4 and 5, but that even the less active of these two would be significantly more active than forum 3.

#### Comparison of Characteristics of Forums

We compared the three forums in phase 1 and the two forums in phase 2 for various metrics. We derived total figures from all activity in phase 1 (447 hours) and phase 2 (1884 hours). To derive standard deviations and 95% confidence intervals, we restricted analysis to time that the forums ran, which was 18 calendar days in phase 1 and 79 calendar days in phase 2 (ie, excluding the first and last partial days and counting the changeover day in phase 2). The four 24-hour metrics were compared between the 3 forums in phase 1 and the 2 forums in phase 2 by analysis of variance (ANOVA), and between those forums in each phase that we perceived to be the quietest (forum 3 in phase 1 and forum 4 in phase 2) using an independent-sample *t* test. As we have made four F tests and four *t* tests, there is minimal scope for familywise error; nevertheless, we report only those results that have *P* ≤ .001.

We also compared the metrics with our views of the forums from our involvement in moderation and in discussion threads, and with views polled from members at the time, and in a subsequent online questionnaire.

#### Identification of Different Patterns of Usage in the Forums

We examined the logging on and posting habits of members, identifying different patterns of online behavior by plotting scattergrams and visually identifying outliers and groups using seven metrics per 24 hours: (1) mean number of episodes, (2) mean number of postings, (3) mean number of topics started, (4) mean number of replies made on other people’s threads, (5) mean number (percentage) of posts made on own thread (ie, a measure of how much participants responded to topics initiated by others compared with how much they were focused on their own topics), (6) total time online, and (7) pages viewed. No statistical tests were carried out.

## Results

### Baseline Description of Participants From Registration Questionnaire

In total, 95 people registered: 77 young people aged 16-25 years (with 47/77 aged under 20) all of whom had self-harmed (YPSH), and 10 health professionals and 8 health care students aged 18-45 years. Among the YPSH, 54 (70%) had self-harmed in the last 4 weeks but four had not self-harmed for more than a year. All 77 had cut themselves at some time. Other frequent forms of self-harm were as follows: not eating (50/77), overdosing (48/77), burning (44/77), biting (35/77), using alcohol or drugs (35/77), binge eating (34/77). Six of the health care professionals had histories of self-harm. All but three participants used the Internet every day.

### Comparison of Forums

#### Comparison of Metrics with Other Data

It was the view of the moderators and researchers during phase 1 that forum 3 was not viable and provided insufficient support for members. These views were largely supported by participants’ views given in a survey in the last few weeks of the study. For example, one survey respondent said “*The earlier groups were a bit too small and resulted in few posts. Meaning you didn’t feel very involved especially if you aren’t confident about making new topics and being very active.”* Moderators, researchers, and participants thought that the reformed forums (4 and 5) in phase 2 were viable and safe. We then asked whether, in retrospect, the metrics confirm the view that forum 3 in the first phase was not viable and, if so, whether these levels could be of use in comparison with other discussion forums.

#### Participation Numbers

There were 95 registered participants who were allocated to three forums in phase 1 and two forums in phase 2. The proportion of inactive participants was higher in phase 2 than in phase 1 as inactivity became cumulative; that is, nearly all participants who did not participate in phase 1 did not participate in phase 2, plus some further participants dropped out. The number of participants, number of active participants, or number of participants who at some time posted did not differ between forums 1,2, and 3 in phase 1, or between forums 4 and 5 in phase 2 (Table 2). We did not look for differences between forum 3 and forum 4.

**Table 2:** Period of study and numbers of participants

		Forum 1	Forum 2	Forum 3	Forum 4	Forum 5
Period of study		5 pm June 15, 2009-8 am July 4, 2009			9:45 am July 4, 2009-9:45 pm September 20, 2009	
Total hours		447	447	447	1884	1884
**People**						
	Registered participants (YPSH^a^)	34	31	30	45	50
	Staff (HCPs^b^)	8	8	8	8	8
**Type of participation**						
	Inactive: participants who never read any messages	3 (9%)	8 (26%)	4 (13%)	17 (38%)	21 (42%)
	Active participants	31 (91%)	23 (74%)	26 (87%)	28 (62%)	29 (58%)
	Participants who never posted any messages	4 (12%)	11 (36%)	12 (40%)	23 (51%)	29 (58%)

^a^ YPSH: young people who self-harm.

^b^ HCPs: health care professionals (National Health Service professionals and final-year students in health/social care disciplines).

#### Episodes

In phase 1, ANOVA showed that the three forums had significantly different total numbers of participant episodes each day (F_2,51_ = 43.3, *P* < .001). The 95% confidence intervals show that forum 3 had fewer participant episodes than forum 2, and forum 2 had fewer than forum 1. But as Table 3 shows (confirmed by a *t* test), forum 3 and phase 2 forum 4 had a similar number of participant episodes.

**Table 3:** Comparison of episode metrics for five discussion forums, including 24-hour metrics (calculated excluding partial calendar days; see methods)

	Forum 1	Forum 2	Forum 3	Forum 4	Forum 5
Total number of participant episodes in study period	1053	761	458	1847	3489
Total number of participant episodes per 24 hours (95% CI^a^)	56.2 (50.8-61.6)	40.1 (36.5-43.7)	23.7 (18.5-29.0)	23.4 (20.4-26.4)	44.2 (40.5-47.9)
Mean number of episodes per participant per 24hours	1.66	1.32	0.82	0.52	0.89
Based on	18 days	18 days	18 days	79 days	79 days
Number of staff episodes	451	451	451	1041	1041
Mean episodes per staff per 24 hours	3.03	3.03	3.03	1.66	1.66

^a^ CI: confidence interval.

#### Time

The total time spent by participants on the discussion forum in 24 hours was less for forum 3 than for forums 1 and 2 (ANOVA: F_2,51_ = 35.2, *P* < .001) but not significantly less than for forum 4 (*t* test) (Table 4).

**Table 4:** Comparison of time metrics for five discussion forums, including 24-hour metrics (calculated excluding partial calendar days; see methods)

	Forum 1	Forum 2	Forum 3	Forum 4	Forum 5
Total participant minutes	24527	15608	4199	23672	53390
Total participant minutes per 24hours (95% CI^a^)	1277 (1042-1512)	820 (647-993)	212 (121-303)	300 (235-365)	676 (558-794)
Mean minutes per participant per 24 hours	38.7	27.0	7.5	6.7	13.6
Based on	18 days	18 days	18 day	79 days	79 days
Staff minutes across forums 1-3 in phase 1 and forums 4-5 in phase 2	13593	13593	13593	21069	21069
Mean minutes per staff per 24 hours across forums 1-3 in phase 1 and forums 4-5 in phase 2	91.2	91.2	91.2	33.5	33.5

^a^ CI: confidence interval.

#### Postings

The total postings each 24 hours was less for forum 3 than for forums 1 and 2 (F_2,51_ = 27.3, *P* < .001) and less than for forum 4 (*t*_81.9_ = -3.3; *P* = .001) (Table 5).

**Table 5:** Comparison of posting metrics for five discussion forums, including 24-hour metrics (calculated excluding partial calendar days; see methods)

	Forum 1	Forum 2	Forum 3	Forum 4	Forum 5
Total participant postings	793	1469	198	1797	1784
Total participant postings per 24hours (95% CI^a^)	36.6 (28.9-44.3)	75.9 (56.0-95.8)	9.3 (5.0-13.6)	22.0 (15.9-28.2)	21.6 (17.1-26.1)
Based on	18 days	18 days	18 days	79 days	79 days
Mean postings per participant	23.3	47.4	6.6	39.9	35.7
Mean postings per participants who read any pages	25.6	63.9	7.6	64.2	61.5
Total staff postings across forums 1-3 in phase 1 and forums 4-5 in phase 2	708	708	708	1095	1095
Mean staff postings per 24 hours across forums 1-3 in phase 1 and forums 4-5 in phase 2	38.0	38.0	38.0	13.9	13.9
Mean postings per staff member in phase 1 (forums 1-3) and phase 2 (forums 4-5)	88.5	88.5	88.5	136.9	136.9

^a^ CI: confidence interval.

#### Reading

The number of pages viewed by all participants in 24 hours in forum 3 was less than in forums 1 and 2 (F_2,5_ = 21.4, *P* < .001) but, with this sample size, not quite significantly less than in forum 4 (Table 6).

**Table 6:** Comparison of reading metrics for five discussion forums, including 24-hour metrics (calculated excluding partial calendar days; see methods)

	Forum 1	Forum 2	Forum 3	Forum 4	Forum 5
Pages viewed by participants	26844	25906	5378	36022	71488
Mean participant page views per 24 hours (95% CI^a^)	1378 (1095-1661)	1352 (996-1708)	265 (164-366)	456 (350-562)	909 (741-1077)
Based on	18 days	18 days	18 days	79 days	79 days
Mean participant page views per participant	790	836	179	800	1430
Pages viewed by staff across forums 1-3 (phase 1) and forums 4-5 (phase 2)	20237	20237	20237	30226	30226
Mean staff page views per 24 hours across forums 1-3 (phase 1) and forums 4-5 (phase 2)	1087	1087	1087	385	385
Mean staff page views across forums 1-3 (phase 1) and forums 4-5 (phase 2)	2530	2530	2530	3778	3778

^a^ CI: confidence interval.

Overall forum metrics may mask where within a forum activity is taking place. Table 7 shows in which rooms postings were made, showing that forum 2 had a very lively random/off-topic room. This table also offers evidence that the change in forums was beneficial. Staff postings in the first period made up 29% (260/894) of postings for support and 18% (556/3013) of all postings. In phase 2, staff postings made up a smaller proportion of support postings (425/1746, 24%) but a slightly greater proportion of all postings (960/4541, 21%). This suggests (a view expressed by moderators) that the reconfigured forums required less intensive input from moderators but that they then joined in elsewhere in the forums. This has implications in terms of the size of forums that are designed to provide meaningful support to participants.

**Table 7:** Total postings in discussion, off-topic, and support rooms (excludes some postings to a general room by participants and postings to the moderator room by staff)

Room	Phase 1					Phase 2			
	Forum 1	Forum 2	Forum 3	Staff	Total	Forum 4	Forum 5	Staff	Total
Discussion	270	612	86	206	1174	312	471	338	1121
Off-topic	127	709	19	90	945	799	678	197	1674
Support	396	147	91	260	894	686	635	425	1746
All	793	1468	196	556	3013	1797	1784	960	4541

Both off-topic rooms were quite active in phase 2, with the most posts on any one thread being 267 on “the or game part 2” (a game played by participants) in group 4, running from July 4, 2009 to August 5, 2009. In total, 46/424 posts or threads (11%) had no replies; that is, they were threads of the first post only (this includes threads in the moderator room). In total, 35% (147/424) of threads were started by the research team and 65% (277/424) by the participants.

Metrics per participant are perhaps less useful for comparing forums because of the impact of denominators. A number of participants who only read a few messages in the first phase then dropped out, so that the proportion of nonparticipants in forums 4 and 5 (phase 2) was higher (38% and 42%) than in the three forums in phase 1 (9%, 13%, and 26%, respectively). However, we have used participant-based metrics to identify participative stances within forums.

### Participative Stances in the Discussion Forums

Participant-based metrics were used to identify outliers and specific types of participants. These participants were named as follows.

#### Caretaker

**Figure 1:**
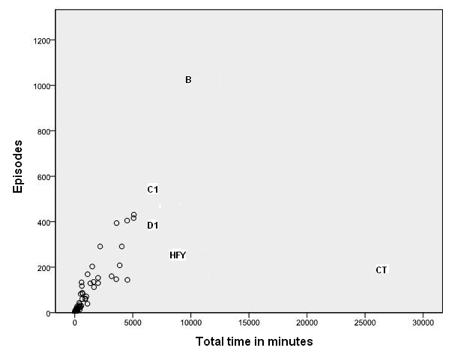
Number of episodes versus total time logged on to the discussion forum for all participants. This shows Caretaker (CT) (with over 25,000 minutes logged on) and Butterfly (B) (with many short episodes) as outliers, as well as Discussant 1 (D1), Crisis-oriented 1 (C1), and Here for you (HFY)

**Figure 2:**
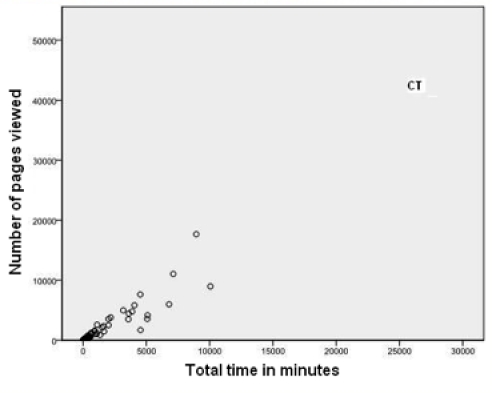
Number of pages viewed versus total time in minutes for all participants. This shows the Caretaker (CT) (with over 25,000 minutes logged on and over 40,000 pages viewed) as an outlier

**Figure 3:**
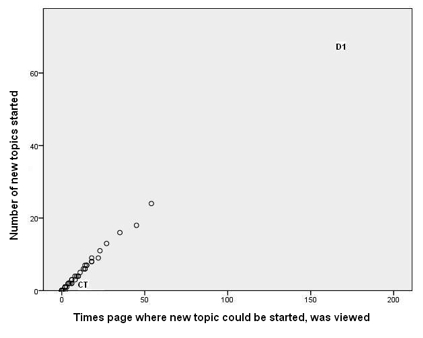
Number of topics started by each participant versus number of times they viewed the page from where new topics could be started. This shows two outliers: Discussant 1 (D1), who started many topics, and Caretaker (CT)

**Figure 4:**
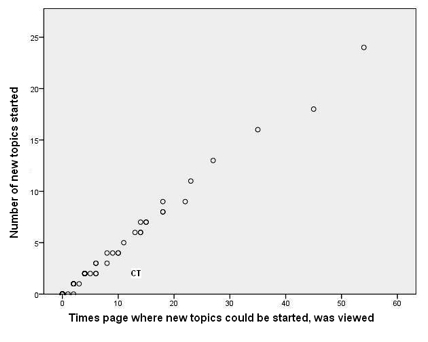
Detail from Figure 3, showing Caretaker (CT) as outlier, having viewed the page from were new topics are started many more times than she or he started new topics

Figures 1 and 2  show that one person (marked CT on both figures) was logged on for a much greater time than most other participants. This person had relatively few episodes but was logged on for very long periods of time. While logged on, she or he reviewed numerous pages but, given the amount of time online, posted fairly infrequently. This person had all of the 32 longest episodes. CT did post, but rarely started topics. Figures 3 and 4 illustrate well this person’s online behavior: CT viewed the page to start a new topic 13 times but started only two new topics. For the other 81/95 participants who viewed at least one page, the mean time per episode ranged from 2 to 36 minutes, whereas CT had a mean time per episode of 134 minutes. In total, the 82 participants had 7611 episodes, 17% (1330/7611) of which were 1 minute or less, but with a long tail going to 1254 minutes (21 hours). We named this type of use of the forum as *Caretaker* to suggest being always around and being watchful, participating to some degree but not initiating many threads. The Caretaker was in forum 1 and then forum 5.

#### Butterfly

**Figure 5:**
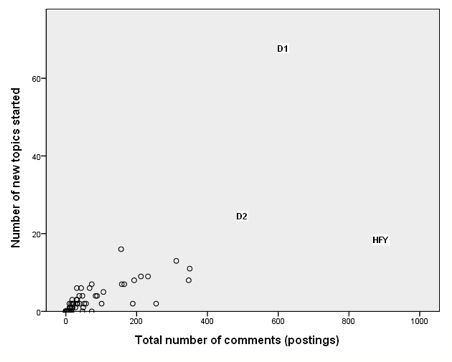
Number of topics started versus total number of comments (postings) showing Discussant 1 (D1), Discussant 2 (D2), and Here for you (HFY) as outliers

The person we characterised as *Butterfly* (marked B on Figure 5) spent a lot of time online but his or her main characteristic was the large number of episodes (1024), with a relatively short mean episode length of 10 minutes. That is, this person’s usage pattern was to log on very frequently, have a quick look around, and log off again. Butterfly was in forum 3 and then forum 5.

#### Discussant

**Figure 6:**
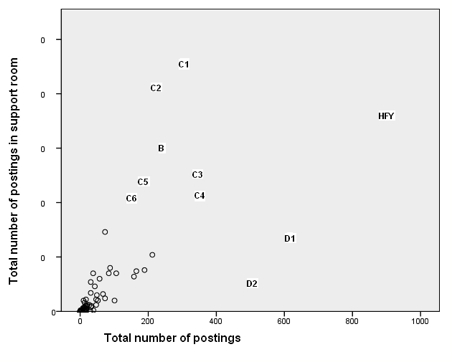
Total number of comments (postings) made in the support room versus total number of postings, showing Here for you (HFY), Discussants 1 and 2 (D1 and D2), Butterfly (B), and Crisis-oriented 1-6 (C1 to C6)

**Figure 7:**
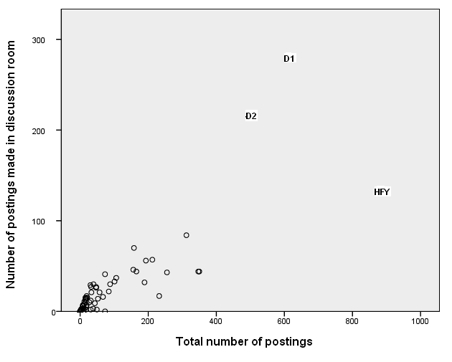
Comments (postings) made in the discussion room versus total number of postings, showing Discussants 1 and 2 (D1 and D2) and Here for you (HFY) as outliers

This stance was adopted by two people (marked D1 and D2 on Figures 5). D1 initiated many more discussion threads than anybody else. Although not as extreme as D1, D2 also initiated a large number of threads but dropped out of *SharpTalk* before the end of the project. The *Discussants* posted proportionately less in the support room (Figure 6) and more in the discussion room (Figure 7). D1 was in forum 2 and then forum 5. D2 was in forum 2 and then forum 4.

#### Here For You

**Figure 8:**
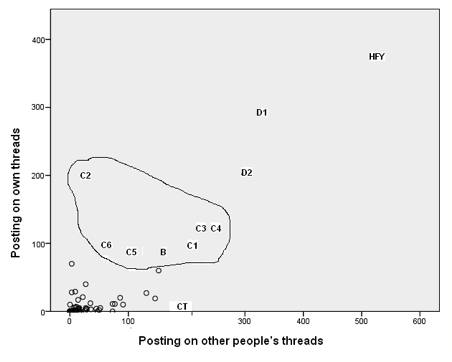
Number of postings on their own threads versus postings on other people’s threads showing Here for you (HFY), Discussants 1 and 2 (D1 and D2), Butterfly (B), Caretaker (CT), and Crisis-oriented 1-6 (C1 to C6)

The person marked HFY (*Here for you*) on Figure 8 posted the most comments but initiated far fewer threads. HFY posted a lot in the support room in response to other participants’ threads. HFY was in forum 2 and then forum 4.

#### Crisis-Oriented Individuals

Figure 6 shows seven people (C1, C2, B [Butterfly], C3, C4, C5, C6) who were crisis oriented insofar as most of their posting activity took place in the support room. These same seven people are shown on Figure 8. These simple metrics do not allow us to see whether these were people in crisis who were requesting support or were responding to others’ distress. However, we see that one individual in particular (C2) posted mainly on their own threads and relatively infrequently on those of others. In comparison, we can see Caretaker (CT), who posted nearly 200 comments but started only three threads in the support room. Our knowledge of the actual content of these postings confirms that these seven people were often in crisis.

## Discussion

How useful is analysis of various metrics from log data in helping to understand and describe the characteristics of an online community? Can such metrics, as an additional method to qualitative methods, help us compare one discussion forum with another, and do they have validity when compared with other methods of forum analysis such as online surveys, and thematic or discourse analysis? Do they tell us anything new about the ways in which participants behave in a forum – to use Strijbos’s term, their “participative stance” [17-20]? If so, should metrics be further developed and used to allow moderators and forum owners to monitor and adapt their forums in real time?

Our study suggests that the routine provision of metrics to owners and moderators of discussion forums could help them in two ways.

### Comparison of Forums

Metrics could provide a second opinion as to whether action is needed to change a forum that is not working well, or guidance on target recruitment numbers for a sustainable forum. We made a judgment, based on the postings that we saw, that forum 3 was not safe as a support community, whereas all other forums were providing adequate support for members. We might hypothesize, from our experience, that similar forums to *SharpTalk*, with less than 300 participant minutes, fewer than 15 participant postings, or fewer than 300 participant page views per 24 hours, are likely to be too small to be viable.

Others setting up small discussion forums with the intention of using them as support groups or online focus groups need to estimate how many participants are needed to make them viable. In face-to-face focus groups, group sizes of 6-8 are typical. Online focus groups are likely to need many more, perhaps 35-40 active members, but further work based on metrics of traffic would be worthwhile as a guide.

Clearly our one case study may be atypical in terms of its participants and activity, but if metrics that seem to distinguish between failing and viable forums were routinely available for more forums, they would provide guidance on whether some change to a forum is needed. These metrics will depend on the functions of the forum. In an educational setting, for example, small group learning may suffer from forums being too big, and numbers of 3-6 may be more appropriate [25].

### Managing or Moderating a Forum

In face-to-face focus or therapeutic groups, the facilitator can watch the body language of the participants and can identify individuals who need extra help or encouragement to be drawn into the discussion. Metrics may help in trying to plug that gap but will only be of use if they are available in real time. They could provide contextual information to moderators of online focus groups who may wish to take action on the basis of the participative stances of members, as judged by metrics, where the actual postings may not tell the whole story.

In online collaborative learning, for example, Strijbos and De Laat [19] recently described various participative stances. They reviewed the literature on classifying online learners and published their own ideas, including nametags such as Captain, Over-rider, Free-rider, Ghost, Pillar, Generator, Hanger-on, and Lurker [19]. However, there are two major differences between that strand of work and the current study. First, that work was done with student groups who were task oriented and collaborating on a specific piece of work. While some of the ideas are relevant, it may not be appropriate to use names such as Lurker, which have become disparaging, in the support group setting. Even in the community discussion forum setting, others have challenged the view that Lurkers are “selfish free-riders” [26]. Secondly, and more important, their classifications have been based on transcript analysis rather than metrics.

By exploring the behaviors of outliers, we were able to identify and characterize the participative stances of our members. The categories that we identified may be unique and special to this group of young people, but the approach, if available at the time, could support moderators by giving a fuller picture of forum activity. It is possible that forums will stand a better chance of being successful if they contain certain key characters such as the Discussants and possibly the Caretaker. It is possible that increasing forum size will increase the chance of someone behaving in that way; alternatively, people may be more likely to take on these roles in smaller forums where they perceive their input to make a difference. Both of these statements are conjecture and need further study.

How much is the character of a forum determined by outlying behaviors within the forum? If the Caretaker had been moved to a different forum, would it have changed the dynamic of that forum significantly, or simply changed the average metrics? All participative stances are context dependent but, in our experience, moving the most proactive Discussant (D1) seems likely to have changed the dynamic of the forums, but we have no evidence to support that. Teachers running small group work know which students will work well together from observation. More study is needed of how the same people may take different stances in different forums, but metrics could be calculated and presented in real time, thus offering information that might enable better management of forums.

### Developments Needed

Producing the metrics presented in this paper from the raw data required extensive analysis and data processing. If these metrics are thought to be useful, the implication is that discussion forum software could include the facility to produce metrics to provide rapid feedback. Dimitracopoulou and Bratitsis [27-29] have been developing and evaluating new ways of offering participants, in online learning environments, visualized representations of appropriate interaction analysis indicators in real time, so that they are aware of, and can regulate, their behavior. Such indicators would have been useful for our project, but even simpler approaches than interaction analysis, such as the metrics as presented in our paper, might be useful in many situations.

### Limitations and Generalizability

*SharpTalk* had two major differences from many discussion forums. First, its membership was recruited for a fixed period of study. This is typical of an online focus group [22-24] but not of open discussion forums, in which new members are added to a continuing dialogue. Second, although *SharpTalk* was set up mainly as an online focus group, it also functioned as a *support group* for people with specific health behaviors and needs (self-harm). So the metrics used to compare forums, or at least the values of those metrics, may not be typical of other forums. Similarly, some of the unusual participative stances may not be found frequently in other forums. Nevertheless, the approach, particularly that of plotting scattergrams to identify key outliers, appears generalizable to other online focus groups and worth further study.

This paper is descriptive in that we had hypotheses only about the activity levels in the forums, not the participative stances that we would find. We have conducted eight statistical tests (four ANOVAs and four *t* tests) in this analysis. While this is not a huge number compared with other studies, readers should remember that 1 in 20 statistical tests will be significant at a level of *P* = .05 just by chance alone. In our opinion, the number of statistical test is insufficient to warrant adjustment for multiple testing, and we think it unlikely that chance alone explains all the findings that reached the conventional measure of statistical significance, but it may explain some of them. The robustness of our findings can be tested only by replication by other groups, who will be able to use the findings of the current study to generate testable hypotheses.

### Conclusion

Our post hoc analysis and construction of metrics suggest that (1) by offering an additional way of comparing different discussion forums, metrics may help with their management, and (2) by identifying participative stances of individuals, metrics may allow better moderation and support of forums, and more effective use of the data collected. However, our analysis was time consuming and post hoc, and there was no body of published metrics for other discussion forums. For metrics to be useful, researchers need to publish metrics for their discussion forums and software developers need to offer more real-time metrics facilities. 

## Abbreviations

ANOVA: analysis of variance
HCPs: health care professionals
NHS: National Health Service
YPSH: young people who self-harm
